# Dynamic nomogram for predicting acute kidney injury in patients with acute ischemic stroke: A retrospective study

**DOI:** 10.3389/fneur.2022.987684

**Published:** 2022-09-13

**Authors:** Ganggui Zhu, Zaixiang Fu, Taian Jin, Xiaohui Xu, Jie Wei, Lingxin Cai, Wenhua Yu

**Affiliations:** ^1^Department of Neurosurgery, Hangzhou First People's Hospital, School of Medicine, Zhejiang University, Hangzhou, China; ^2^Department of Neurosurgery, The Second Affiliated Hospital, School of Medicine, Zhejiang University, Hangzhou, China; ^3^Department of Neurosurgery, The Fourth Affiliated Hospital, School of Medicine, Zhejiang University, Yiwu, China

**Keywords:** acute ischemic stroke, acute kidney injury, nomogram, MIMIC-III database, retrospective study

## Abstract

**Background:**

This study sought to develop and validate a dynamic nomogram chart to assess the risk of acute kidney injury (AKI) in patients with acute ischemic stroke (AIS).

**Methods:**

These data were drawn from the Medical Information Mart for Intensive Care III (MIMIC-III) database, which collects 47 clinical indicators of patients after admission to the hospital. The primary outcome indicator was the occurrence of AKI within 48 h of intensive care unit (ICU) admission. Independent risk factors for AKI were screened from the training set using univariate and multifactorial logistic regression analyses. Multiple logistic regression models were developed, and nomograms were plotted and validated in an internal validation set. Based on the receiver operating characteristic (ROC) curve, calibration curve, and decision curve analysis (DCA) to estimate the performance of this nomogram.

**Results:**

Nomogram indicators include blood urea nitrogen (BUN), creatinine, red blood cell distribution width (RDW), heart rate (HR), Oxford Acute Severity of Illness Score (OASIS), the history of congestive heart failure (CHF), the use of vancomycin, contrast agent, and mannitol. The predictive model displayed well discrimination with the area under the ROC curve values of 0.8529 and 0.8598 for the training set and the validator, respectively. Calibration curves revealed favorable concordance between the actual and predicted incidence of AKI (*p* > 0.05). DCA indicates the excellent net clinical benefit of nomogram in predicting AKI.

**Conclusion:**

In summary, we explored the incidence of AKI in patients with AIS during ICU stay and developed a predictive model to help clinical decision-making.

## Introduction

Stroke remains the second most common disease threatening humans worldwide (1). Stroke places a tremendous burden on patients, families, and society, especially in developing countries (2). The American Heart Association and the American Stroke Association project that the total medical cost of stroke will reach $1,841 billion from 2012 to 2030 (3). Ischemic stroke, usually caused by a blood clot blocking an artery in the brain, is the leading type of stroke (4). Tissue plasminogen activator (tPA) is the only drug currently approved by the United States Food and Drug Administration (FDA) for the treatment of ischemic strokes. Patients outside of the time window for tPA use may be treated with other therapies, such as thrombectomy, anticoagulants, blood pressure-lowering, and cholesterol-lowering drugs. Nevertheless, the effectiveness of treatment for ischemic stroke is unsatisfactory. Stroke kills ~6 million people each year, accounting for more than 10% of all deaths (5). Therefore, in the management of ischemic stroke, early identification of patients who may have a poor prognosis and timely intervention is important to improve the quality of patient survival later in life.

Acute kidney injury (AKI) is a common condition in critically ill patients and occurs in ~30–50% of intensive care unit (ICU) patients (6). High mortality is one of its characteristics, with ~20–25% of patients dying during hospitalization (7, 8). AKI manifests itself as a dramatic deterioration of renal function and a decrease in urine output, which leads to water and electrolyte disturbances and high circulating blood volume, resulting in a series of negative effects on other organs (9). After the stroke, activation of the sympathetic nervous system, the renin-angiotensin-aldosterone pathway impairs the self-regulatory function of the kidney (10). Moreover, the immune response induced by stroke leads to a massive release of inflammatory factors, resulting in decreased renal function (11). Therefore, early identification, recognition, and intervention can reduce the probability of AKI and slow down its progression (12).

However, there are no validated clinical models to predict the occurrence of AKI in patients with acute ischemic stroke (AIS). Therefore, there is a need to effectively predict the occurrence of AKI or screen for potential risk factors to guide early clinical intervention to improve prognosis. Based on several key variables and parameters, nomograms, especially dynamic nomograms, provide a powerful and easy-to-use method to predict the outcomes of individual events (13–16). The main objective of this study was to identify factors that independently predict the occurrence of AKI in patients with AIS. The patient cohort and factors were selected from the Medical Information Mart for Intensive Care III (MIMIC-III) database, and the nomogram was concurrently developed to predict the incidence of AKI in the AIS population during ICU (17).

## Materials and methods

### Data source

All data for this study were obtained from a publicly available, large critical care database named MIMIC-III (17). It included 46,520 patients who were admitted to the ICU at Beth Israel Deaconess Medical Center between June 2001 and October 2012. The study was approved by the Institutional Review Board of Beth Israel Deaconess Medical Center and the Massachusetts Institute of Technology. Because data from the MIMIC-III study are publicly available and patient's personal information has been anonymized, the requirement to obtain individual patient consent was waived. After passing an exam called Protecting Human Research Participants (certification number 43391018), we were granted access to the database and extracted data using the structured query language of PostgreSQL 9.6.

### Study patients

Patients admitted with a diagnosis of AIS, defined as the International Classification of Diseases-Ninth (ICD-9) codes 43301, 43311, 43321, 43331, 43381, 43391, 43401, 43411, and 43491, were included in the study population. AKI was diagnosed using The Kidney Disease Improving Global Outcomes (KDIGO) criteria (18): blood creatinine ≥26.5 μmol/l increased within 48 h or increased to 1.5 times the baseline level and urine output <0.5 ml/kg/h for more than 6 h. For patients with multiple admission records, only information on the first ICU admission was retained. The age range was set at 18–89 years because the actual age of patients older than 89 years was not available. People receiving renal replacement therapy or continuous renal replacement therapy and those with a prior history of renal failure or chronic kidney disease were excluded. In addition, patients with an ICU stay of <24 h were also excluded. Ultimately, 1,132 patients were entered into the study and randomized in a 7:3 ratio into the training set (*n* = 792) and validation set (*n* = 340).

### Data extraction

All data were extracted within 24 h of the patient's admission to the ICU. The variables extracted included demographic characteristics, vital signs, interventions, comorbidities, laboratory indicators, and scoring systems (19, 20). Demographic characteristics included age, sex, race, and type of admission. Vital signs measured on admission for the first time were recorded, such as heart rate (HR), respiratory rate (RR), systolic blood pressure (SBP), diastolic blood pressure (DBP), body temperature, and peripheral hemoglobin oxygen saturation (SPO_2_). Interventions, such as vasopressin, mannitol, colloidal injections, aminoglycoside antibiotics, vancomycin, mechanical ventilation, angiotensin-converting enzyme inhibitors (ACEIs), angiotensin receptor blockers (ARBs), intravenous thrombolysis, and endovascular mechanical thrombectomy, were recorded within 24 h of admission. Secondary prevention medications for ischemic stroke were also documented that included antiplatelet agents (e.g., aspirin, clopidogrel, ticlopidine, cilostazol, tegretol, prasugrel, and disulfiram) and anticoagulants (e.g., warfarin, dabigatran, apixaban, rivaroxaban, and edoxaban). Laboratory parameters, such as hemoglobin, white blood cell (WBC), platelet count, blood glucose, sodium, potassium, blood urea nitrogen (BUN), red blood cell distribution width (RDW), prothrombin time (PT), and activated partial thromboplastin time (APTT), recorded in the laboratory event table. In addition, the Oxford Acute Severity of Illness Score (OASIS) and ICU length of stay (ICUlos) were recorded. OASIS is a new score developed using machine-learning algorithms that have been widely used in prognostic prediction models for a range of diseases (21, 22). All comorbidities were identified according to the ICD-9 code records.

### Statistical analysis

**Figure 1:**
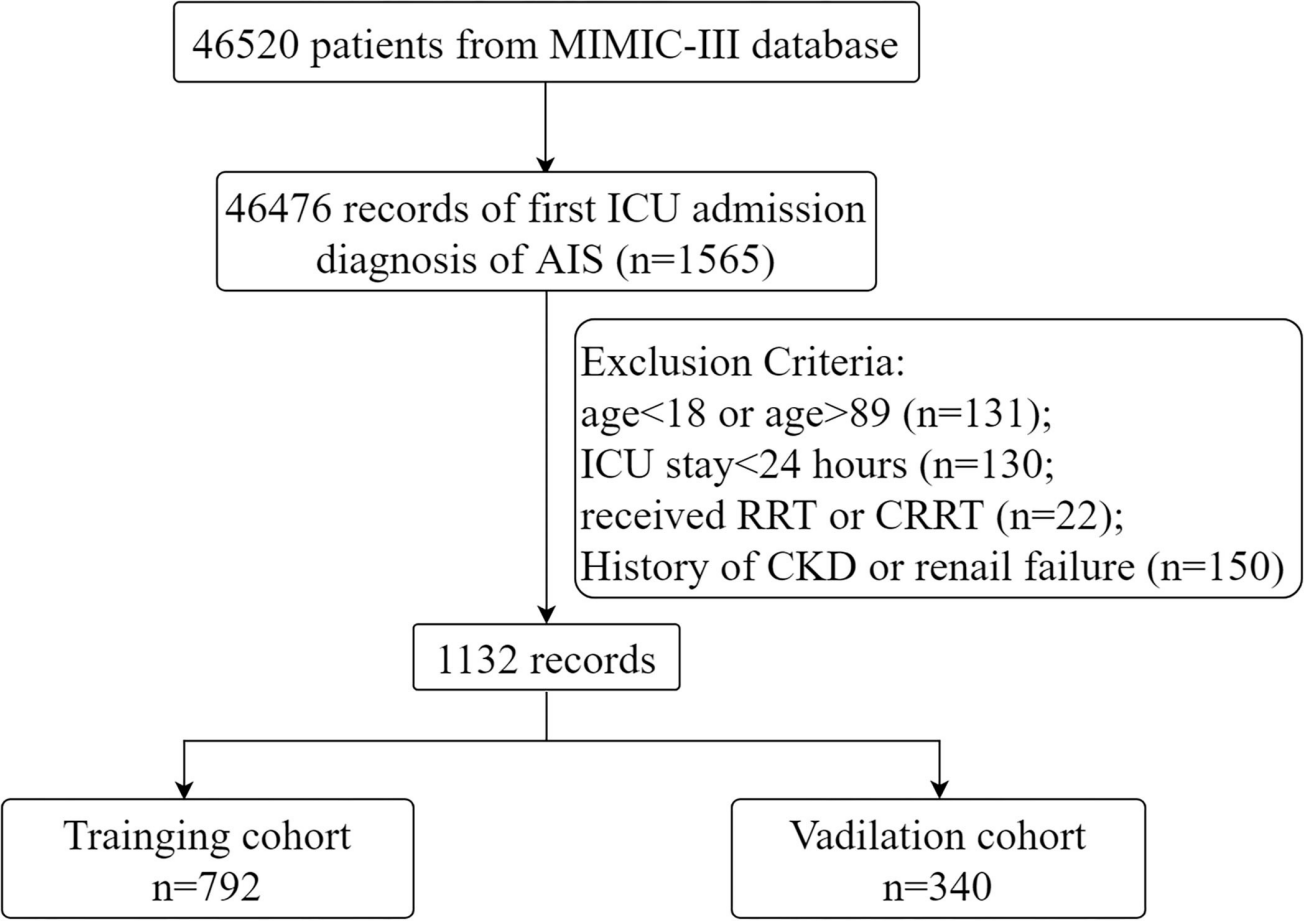
The flowchart of the study.

Continuous variables were compared by *t*-test and Kruskal-Wallis rank sum test, expressed as median and quartiles (Q1 and Q3). Categorical variables were compared using the χ^2^ test or Fisher's exact test. The presence of multicollinearity between covariates was determined by the variance inflation factor (VIF). VIF ≥ 5 was considered to have multicollinearity (23). Univariate logistic analysis was used to assess the association between covariates and outcomes. Variables with *p* < 0.05 were included in the multifactorial regression. In a stepwise backward regression according to the Akaike Information Criterion (AIC), the optimal model holds the minimum AIC value. A nomogram predicting the risk of AKI in patients with AIS was created based on the results of the multifactorial regression. The performance of the nomogram was verified in the validation set using the following methods. The area under the curve (AUC) of the receiver operating characteristic (ROC) curve was used to evaluate the discriminatory ability of the nomogram. Calibration plots and Hosmer-Lemeshow goodness-of-fit tests (HL tests) were used by comparing the accuracy of the nomogram. Decision curve analysis (DCA) was performed to evaluate the clinical applicability of the nomogram. *p* < 0.05 was considered to be statistically significant. All statistical analyses were performed with R statistical software (V.4.1.2) and Stata (V.16.0). The flow chart of the study is shown in Figure 1.

## Results

### Baseline characteristics

The median age of patients with AKI was 72.2 (59.3 and 81.3), older than patients with non-AKI at 68.5 (55.9 and 78.8). The baseline characteristics of all patients are shown in Table 1. In terms of comorbidities, patients with AKI were more likely to have congestive heart failure (CHF), liver disease, coagulopathy, and diabetes than patients with non-AKI. In terms of therapeutic measures received, the AKI group received more aminoglycoside antibiotics, furosemide, vancomycin, antiplatelet agent, and mechanical ventilation as compared to the non-AKI group. In addition, the AKI group received contrast-related tests more frequently, whereas the non-AKI group was more likely to receive intravenous thrombolysis. Furthermore, patients with AKI had higher HR, RR, blood glucose, creatinine, BUN, red blood cell width (RDW), OASIS, and ICUlos and lower SBP, DBP, urine output, and hemoglobin. Comparatively, patients in the non-AKI group usually had higher SBP, DBP, urine output, and hemoglobin. Similar results were obtained in the training set (Table 2). There was no significant difference in the baseline characteristics of the training and validation set populations for each clinical factor as shown in Supplementary material S1. The number of people with AKI was 104 (13.13%) and 48 (14.12%), respectively.

**Table 1:** Characteristics at baseline between acute kidney injury (AKI) and non-AKI groups.

**Variables**	**Total** **(*n* = 1,132)**	**AKI** **(*n* = 152)**	**Non-AKI** **(*n* = 980)**	***P*** **-value**
**Demographic variables**				
Age	69.1 (56.2–79.1)	72.2 (59.3–81.3)	68.5 (55.9–78.8)	0.02
Gender				0.457
Female	568 (50.2%)	72 (47.4%)	496 (50.6%)	
Male	564 (49.8%)	80 (52.6%)	484 (49.4%)	
Ethnicity				0.071
White	814 (71.9%)	100 (65.8%)	714 (72.9%)	
Non-white	318 (28.1%)	52 (34.2%)	266 (27.1%)	
Admission–type				0.259
Emergency/urgent	1,047 (92.5%)	144 (94.7%)	903 (92.1%)	
Elective	85 (7.5%)	8 (5.3%)	77 (7.9%)	
**Comorbidities**				
Hypertension	690 (61.0%)	97 (63.8%)	593 (60.5%)	0.437
CHF	235 (20.8%)	64 (42.1%)	171 (17.4%)	<0.001
Peripheral vascular	116 (10.2%)	16 (10.5%)	100 (10.2%)	0.903
Chronic pulmonary	185 (16.3%)	28 (18.4%)	157 (16.0%)	0.456
Liver disease	55 (4.9%)	23 (15.1%)	32 (3.3%)	<0.001
Rheumatoid arthritis	32 (2.8%)	7 (4.6%)	25 (2.6%)	0.155
Coagulopathy	79 (7.0%)	32 (21.1%)	47 (4.8%)	<0.001
Obsity	43 (3.8%)	7 (4.6%)	36 (3.7%)	0.576
Alcohol abuse	74 (6.5%)	13 (8.6%)	61 (6.2%)	0.28
Diabetes	306 (27.0%)	57 (37.5%)	249 (25.4%)	0.002
**Treatment measures**				
Vasopressin	136 (12.0%)	41 (27.0%)	95 (9.7%)	<0.001
Mannitol	159 (14.0%)	9 (5.9%)	150 (15.3%)	0.002
Colloid bolus	82 (7.2%)	15 (9.9%)	67 (6.8%)	0.18
ACEI/ARBs	133 (11.7%)	23 (15.1%)	110 (11.2%)	0.164
Vancomycin	68 (6.0%)	16 (10.5%)	52 (5.3%)	0.012
Furosemide	498 (44.0%)	103 (67.8%)	395 (40.3%)	<0.001
Glycopeptide antibiotics	456 (40.3%)	94 (61.8%)	362 (36.9%)	<0.001
Mechanical Ventilation	352 (31.1%)	58 (38.2%)	294 (30.0%)	0.043
Antiplatelet	869 (76.8%)	129 (84.9%)	740 (75.5%)	0.011
Anticoagulation	412 (36.40%)	58 (38.16%)	354 (36.12%)	0.627
Contrast agents	956 (84.4%)	136 (89.5%)	820 (83.6%)	0.012
Intravenous thrombolysis	786 (69.4%)	78 (51.3%)	708 (72.2%)	<0.001
Endovascular mechanical thrombectomy	245 (21.6%)	40 (26.3%)	205 (20.9%)	0.075
**Laboratory test**				
RR, breaths/min	18.2 (16.4–20.8)	19.9 (17.5–23.6)	18.1 (16.2–20.3)	<0.001
HR, beats/min	80.7 (70.4–92.0)	90.6 (77.7–105.0)	79.6 (69.6–89.4)	<0.001
SBP, mmHg	130.5 (115.6–143.9)	119.1 (108.6–136.5)	131.5 (116.8–144.5)	<0.001
DBP, mmHg	64.4 (56.6–72.5)	61.6 (55.1–72.7)	64.8 (57.0–72.5)	0.046
Temperature, °C	36.9 (36.6–37.3)	36.9 (36.5–37.3)	36.9 (36.6–37.3)	0.919
SPO2,%	97.8 (96.6–99.0)	97.6 (96.2–98.7)	97.9 (96.7–99.0)	0.039
Urine output, ml	1770.0 (1148.8–2487.5)	1517.5 (863.8–2132.5)	1843.5 (1175.0–2525.2)	<0.001
WBC, K/uL	10.2 (7.8–13.1)	10.2 (7.8–14.7)	10.2 (7.8–12.9)	0.464
Hemoglobin, g/dl	11.3 (9.9–12.9)	10.4 (9.2–11.4)	11.5 (10.1–13.0)	<0.001
Platelets, K/uL	239.0 (182.0–318.2)	223.5 (153.5–303.8)	243.0 (185.8–320.0)	0.021
Blood glucose, mg/dl	121.5 (104.0–148.2)	123.0 (105.0–150.2)	121.0 (103.0–147.0)	0.319
Sodium, mEq/L	139.0 (137.0–142.0)	139.0 (136.0–142.0)	139.0 (137.0–142.0)	0.165
Potassium, mEq/l	3.9 (3.7–4.3)	4.0 (3.7–4.4)	3.9 (3.6–4.3)	0.125
Creatinine, mg/dl	0.8 (0.7–1.1)	1.2 (0.9–1.5)	0.8 (0.6–1.0)	<0.001
BUN, mg/dl	18.0 (13.0–25.0)	26.0 (18.0–41.2)	17.0 (12.0–24.0)	<0.001
RDW	14.1 (13.4–15.3)	15.1 (14.1–16.5)	14.0 (13.3–15.1)	<0.001
PT, seconds	13.6 (12.7–15.4)	14.5 (13.4–17.2)	13.5 (12.6–15.1)	<0.001
APTT, seconds	29.8 (25.4–40.3)	32.8 (27.5–43.3)	29.4 (25.2–39.8)	0.016
OASIS	33.0 (27.0–39.0)	37.0 (31.0–43.0)	33.0 (26.0–39.0)	<0.001
ICUlos, hours	76.0 (44.0–169.0)	113.0 (60.2–283.5)	72.0 (43.0–163.0)	<0.001

CHF, congestive heart failure; ACEI, angiotensin-converting enzyme inhibitors; ARBs, angiotensin receptor blockers; RR, respiratory rate; HR, heart rate; SBP, systolic blood pressure; DBP, diastolic blood pressure; MBP, mean blood pressure; SpO_2_, percutaneous oxygen saturation; WBC, white blood cell; BUN, blood urea nitrogen; PT, prothrombin time; RDW, red blood cell width; PT, prothrombin time; APTT, activated partial thromboplastin time; OASIS, Oxford Acute Severity of Illness Score; ICUlos, intensive care unit length of stay.

**Table 2:** Characteristics at baseline between AKI and non-AKI in train group.

**Variables**	**AKI** **(*n* = 104)**	**Non-AKI** **(*n* = 688)**	***P*** **-value**
Age	73.0 (62.2–81.1)	68.1 (56.1–78.6)	0.009
Gender			0.942
Female	51 (49.0%)	340 (49.4%)	
Male	53 (51.0%)	348 (50.6%)	
Ethnicity			0.082
White	67 (64.4%)	500 (72.7%)	
Non-white	37 (35.6%)	188 (27.3%)	
Admission-type			0.526
Emergency/urgent	97 (93.3%)	629 (91.4%)	
Elective	7 (6.7%)	59 (8.6%)	
**Comorbidities**			
Hypertension	64 (61.5%)	427 (62.1%)	0.918
CHF	41 (39.4%)	123 (17.9%)	<0.001
Peripheral vascular	8 (7.7%)	72 (10.5%)	0.382
Chronic pulmonary	21 (20.2%)	108 (15.7%)	0.247
Liver disease	13 (12.5%)	25 (3.6%)	<0.001
Rheumatoid arthritis	4 (3.8%)	17 (2.5%)	0.416
Coagulopathy	25 (24.0%)	34 (4.9%)	<0.001
Obsity	2 (1.9%)	26 (3.8%)	0.339
Alcohol abuse	10 (9.6%)	46 (6.7%)	0.277
Diabetes	39 (37.5%)	175 (25.4%)	0.01
**Treatment measures**			
Vasopressin	24 (23.1%)	62 (9.0%)	<0.001
Mannitol	5 (4.8%)	102 (14.8%)	0.005
Colloid bolus	10 (9.6%)	51 (7.4%)	0.432
ACEI/ARBs	17 (16.3%)	80 (11.6%)	0.171
Vancomycin	65 (62.5%)	251 (36.5%)	<0.001
Furosemide	70 (67.3%)	286 (41.6%)	<0.001
Glycopeptide antibiotics	10 (9.6%)	33 (4.8%)	0.043
Mechanical Ventilation	41 (39.4%)	199 (28.9%)	0.03
Antiplatelet	85 (81.7%)	521 (75.7%)	0.178
Anticoagulation	43 (41.3%)	256 (37.2%)	0.417
Contrast agents	92 (88.5%)	581 (84.4%)	0.018
Intravenous thrombolysis	53 (52.4%)	492 (71.5%)	0.005
Endovascular mechanical thrombectomy	26 (25.6%)	148 (21.5%)	0.126
**Laboratory test**			
RR, breaths/min	19.9 (17.1–23.3)	18.0 (16.2–20.1)	<0.001
HR, beats/min	88.9 (77.6–103.7)	79.4 (69.4–88.8)	<0.001
SBP, mmHg	119.1 (108.5–137.4)	130.8 (116.7–144.6)	0.002
DBP, mmHg	62.3 (53.7–72.9)	65.0 (56.8–72.5)	0.144
Temperature, °C	36.8 (36.5–37.2)	36.9 (36.6–37.3)	0.589
SPO_2_,%	97.9 (96.5–98.8)	97.9 (96.7–99.1)	0.37
Urine output, ml	1486.5 (911.2–2140.0)	1836.0 (1169.2–2529.5)	0.002
WBC, K/uL	10.4 (8.0–15.5)	10.2 (7.7–12.9)	0.184
Hemoglobin, g/dl	10.2 (9.3–11.1)	11.5 (10.1–13.0)	<0.001
Platelets, K/uL	224.5 (152.0–311.0)	239.0 (183.0–311.2)	0.15
Blood glucose, mg/dl	125.0 (105.0–157.2)	121.0 (103.0–147.0)	
Sodium, mEq/L	139.0 (135.0–142.0)	139.0 (138.0–142.0)	0.248
Potassium, mEq/l	4.0 (3.6–4.3)	3.9 (3.7–4.3)	0.426
Creatinine, mg/dl	1.2 (0.9–1.5)	0.8 (0.6–1.0)	<0.001
BUN, mg/dl	24.5 (18.0–39.0)	17.0 (12.0–24.0)	<0.001
RDW	15.2 (14.1–16.4)	14.0 (13.3–15.0)	<0.001
PT, seconds	14.2 (13.3–16.6)	13.5 (12.6–15.1)	0.005
APTT, seconds	33.0 (27.1–43.8)	29.9 (25.1–40.0)	0.042
OASIS	37.0 (31.0–41.0)	32.5 (26.0–38.0)	<0.001
ICUlos, hours	109.0 (65.5–266.0)	73.0 (43.0–158.2)	<0.001

CHF, congestive heart failure; ACEI, angiotensin-converting enzyme inhibitors; ARBs, angiotensin receptor blockers; RR, respiratory rate; HR, heart rate; SBP, systolic blood pressure; DBP, diastolic blood pressure; MBP, mean blood pressure; SpO_2_, percutaneous oxygen saturation; WBC, white blood cell; BUN, blood urea nitrogen; PT, prothrombin time; RDW, red blood cell width; PT, prothrombin time; APTT, activated partial thromboplastin time; OASIS, Oxford Acute Severity of Illness Score; ICUlos, intensive care unit length of stay.

### Variables selection and nomogram construction

**Figure 2:**
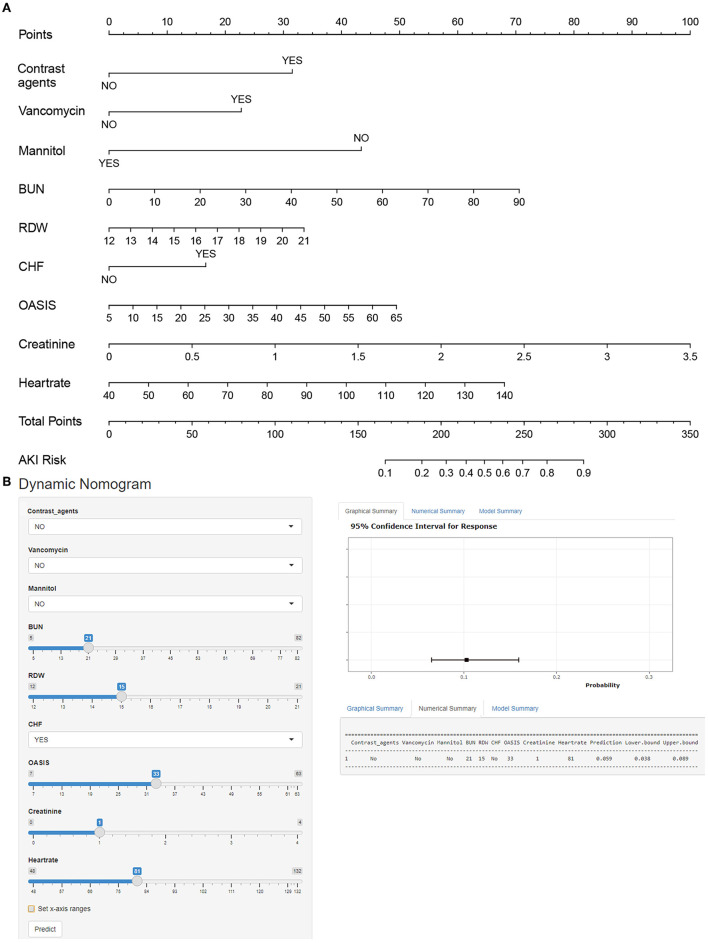
**(A)** Nomogram to identify the risk of acute kidney injury (AKI) in patients with AIS, based on logistic regression analysis. To acquire the corresponding scores for each variable, draw a vertical line upward to the “Points” axis. Sum the score for all predictors and locate the final value on the “Total Points” axis. Draw a line straight down to the “Risk” axis to determine the risk of AKI. **(B)** Dynamic nomogram line graph interface. BUN, blood urea nitrogen; RDW, red blood cell width; CHF, congestive heart failure; OASIS, Oxford Acute Severity of Illness Score.

Multiple covariance tests revealed no significant covariance among the variables as shown in Supplementary material S2, so all variables were included in the univariate logistic regression. Based on the univariate regression results, 29 variables were significantly associated with the occurrence of AKI in patients with AIS (Table 3). Then, based on AIC, 9 factors were selected: BUN, creatinine, RDW, HR, the use of vancomycin, contrast agent, mannitol agent, the history of CHF, and OASIS. Multivariate logistic regression analysis showed risk factors that were independently associated with the development of AKI in patients with AIS (Table 4). A model was constructed using the above 9 factors, on the basis of which a nomogram was constructed to predict the risk of AKI in patients with AIS (Figure 2). In addition, the dynamic nomogram can be accessed through https://dynnomo.shinyapps.io/PREDICTAKI/.

**Table 3:** Univariate logistic regression analysis of predictive variables of acute kidney injury (AKI) in the training cohort.

**Variables**	**OR**	**95%CI**	***P***
Age	1.032	(1.02–1.04)	0.000
Gender (male)	0.893	(0.83–0.96)	0.001
Ethnicity (White)	0.811	(0.61–1.07)	0.143
Admission-type (emergency/urgent)	0.817	(0.49–1.36)	0.439
Hypertension	1.228	(0.94–1.61)	0.133
Congestive heart failure	0.602	(0.44–0.83)	0.002
Peripheral vascular	0.998	(1.00–1.00)	0.000
Chronic pulmonary	0.421	(0.23–0.76)	0.004
Liver disease	1.208	(0.80–1.82)	0.363
Rheumatoid arthritis	1.051	(1.04–1.06)	0.000
Coagulopathy	0.365	(0.20–0.67)	0.001
Obesity	0.875	(0.55–1.40)	0.576
Alcohol abuse	1.314	(0.76–2.26)	0.325
Diabetes	0.340	(0.25–0.46)	0.000
Vasopressin	0.849	(0.54–1.34)	0.479
Mannitol	0.538	(0.39–0.74)	0.000
Colloid bolus	0.631	(0.44–0.90)	0.011
ACEI/ARBs	0.351	(0.22–0.57)	0.000
Aminoglycoside antibiotics	1.406	(0.76–2.60)	0.279
Furosemide	1.007	(1.00–1.01)	0.000
Vancomycin	1.014	(1.00–1.02)	0.003
Mechanical ventilation	0.907	(0.65–1.26)	0.561
Antiplatelet	0.316	(0.13–0.79)	0.013
Anticoagulation	0.916	(0.64–1.30)	0.628
Contrast agents	0.176	(0.11–0.32)	0.000
Intravenous thrombolysis	0.386	(0.25–0.60)	0.000
Endovascular mechanical thrombectomy	2.097	(0.69–3.58)	0.110
RR, breaths/min	2.129	(1.50–3.03)	0.000
HR, beats/min	1.052	(1.03–1.08)	0.000
SBP, mmHg	1.006	(0.97–1.04)	0.737
DBP, mmHg	1.154	(0.85–1.57)	0.365
Temperature, °C	0.999	(1.00–1.00)	0.000
SPO_2_,%	1.227	(0.97–1.56)	0.092
Urine output, ml	0.602	(0.45–0.80)	0.001
WBC, K/uL	1.040	(1.01–1.07)	0.007
Hemoglobin, g/dl	0.690	(0.52–0.92)	0.012
Platelets, K/uL	1.010	(0.98–1.04)	0.509
Blood glucose, mg/dl	1.041	(1.03–1.06)	0.000
Sodium, mEq/L	0.997	(0.99–1.00)	0.446
Potassium, mEq/l	1.002	(0.99–1.01)	0.595
Creatinine, mg/dl	0.993	(0.98–1.00)	0.005
BUN, mg/dl	0.778	(0.53–1.14)	0.000
RDW	1.330	(0.56–3.17)	0.010
PT, seconds	1.102	(1.01–1.20)	0.027
APTT, seconds	1.055	(1.02–1.09)	0.005
OASIS	1.051	(1.03–1.07)	0.000

CHF, congestive heart failure; ACEI, angiotensin-converting enzyme inhibitors; ARBs, angiotensin receptor blockers; RR, respiratory rate; HR, heart rate; SBP, systolic blood pressure; DBP, diastolic blood pressure; MBP, mean blood pressure; SpO_2_, percutaneous oxygen saturation; WBC, white blood cell; BUN, blood urea nitrogen; PT, prothrombin time; RDW, red blood cell width; PT, prothrombin time; APTT, activated partial thromboplastin time; OASIS, Oxford Acute Severity of Illness Score.

**Table 4:** Multivariate logistic regression analysis of candidate factors of acute kidney injury (AKI) in the training cohort.

**Variables**	**β**	***P*** **-value**	**OR**	**95%CI**
BUN	0.029	0.000	1.029	(1.013–1.046)
Creatinine	1.051	0.000	2.860	(1.845–4.434)
Contrast agent	1.160	0.001	3.189	(1.580–6.434)
RDW	0.137	0.049	1.147	(1.000–1.315)
HR	0.025	0.002	1.025	(1.009–1.042)
Vancomycin	0.837	0.001	2.308	(1.407–3.788)
CHF	0.612	0.020	1.844	(1.100–3.093)
OASIS	0.030	0.038	1.031	(1.002–1.061)
Mannitol	−1.596	0.006	0.203	(0.065–0.628)

BUN, blood urea nitrogen; CHF, congestive heart failure; HR, heart rate; OASIS, Oxford Acute Severity of Illness Score; RDW, red blood cell width.

### Nomogram validation

**Figure 3:**
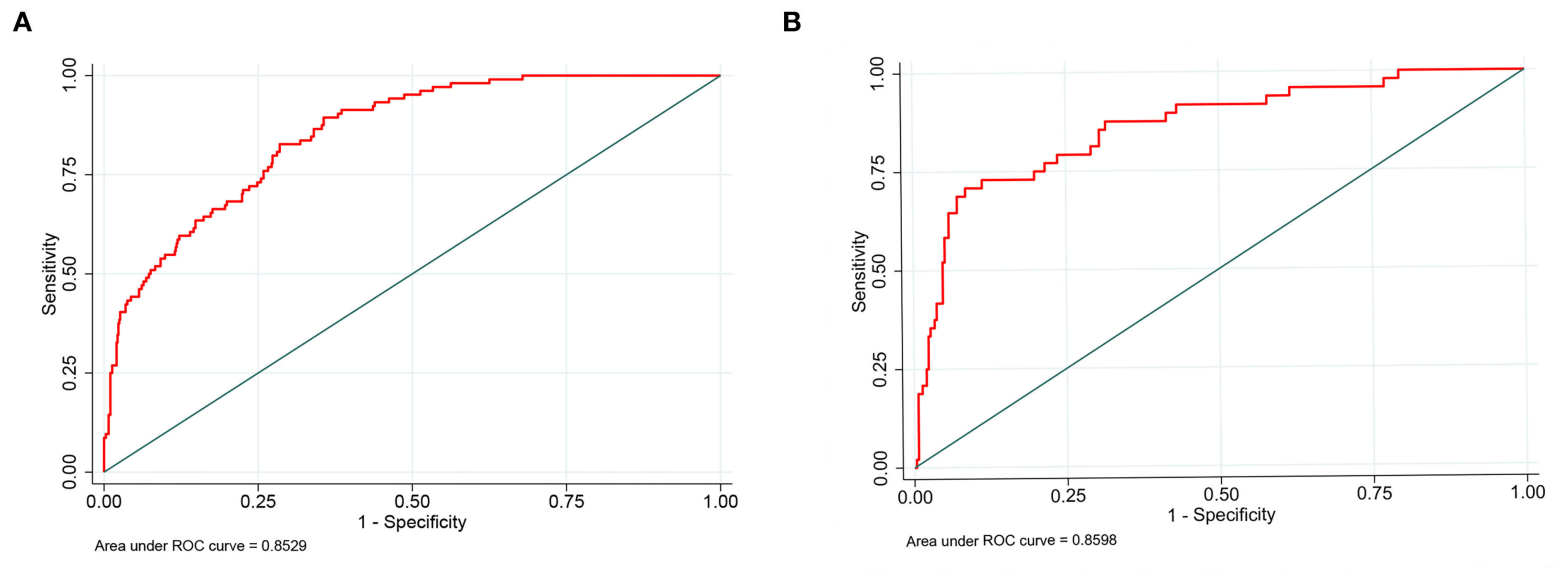
Receiver operating characteristic curve of the nomogram. The an area under the curve (AUC) of the nomogram for the prediction of acute kidney injury (AKI) in patients with AIS was 0.8529 in the training set **(A)** and 0.8598 in the validation set **(B)**.

**Figure 4:**
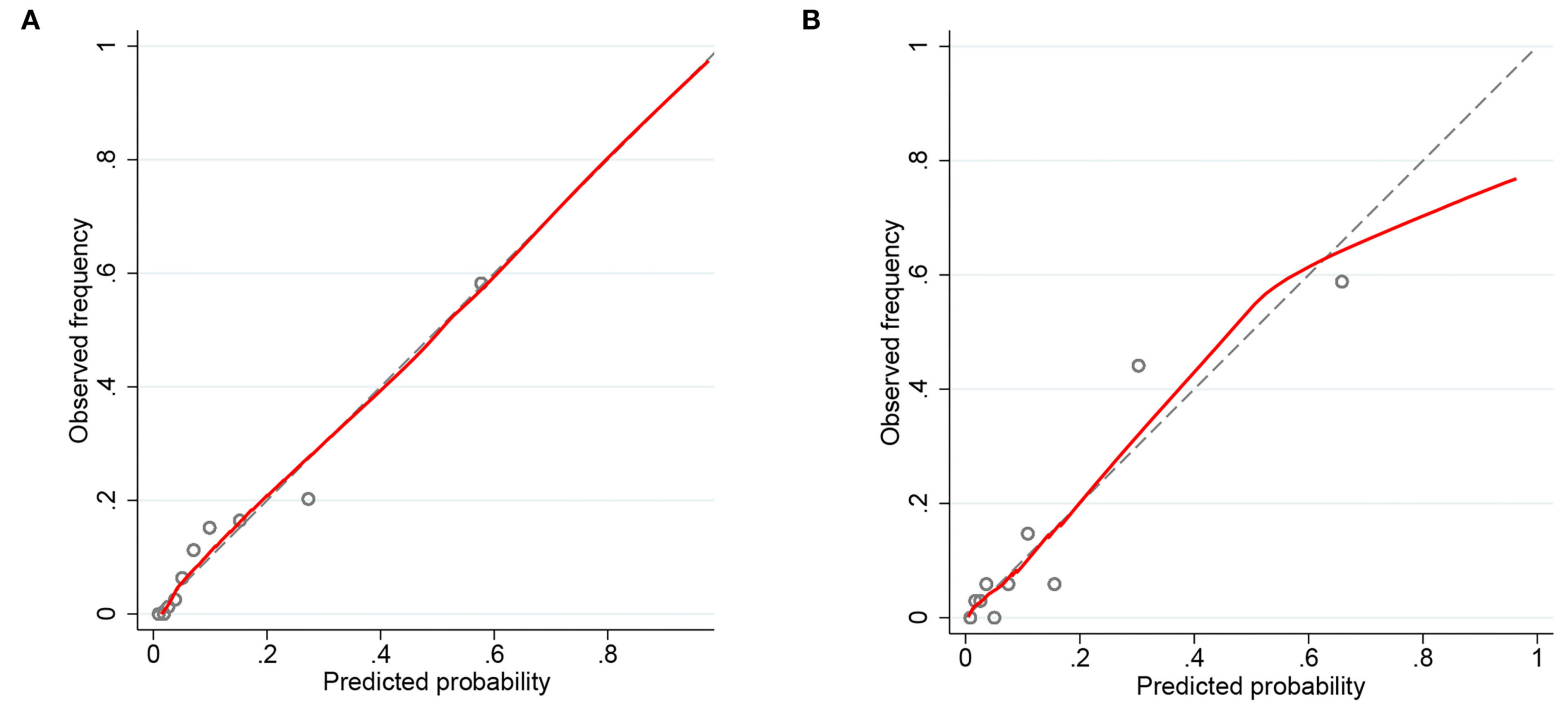
Calibration curves of the predicted nomogram in the training set **(A)** and validation set **(B)**. The x-axis represents the predicted probability calculated by the nomogram, and the y-axis is the observed actual probability of acute kidney injury (AKI). Results of the Hosmer-Lemeshow test demonstrate that the *p*-value of the training set is 0.8814 and the validation set is 0.4835, respectively.

**Figure 5:**
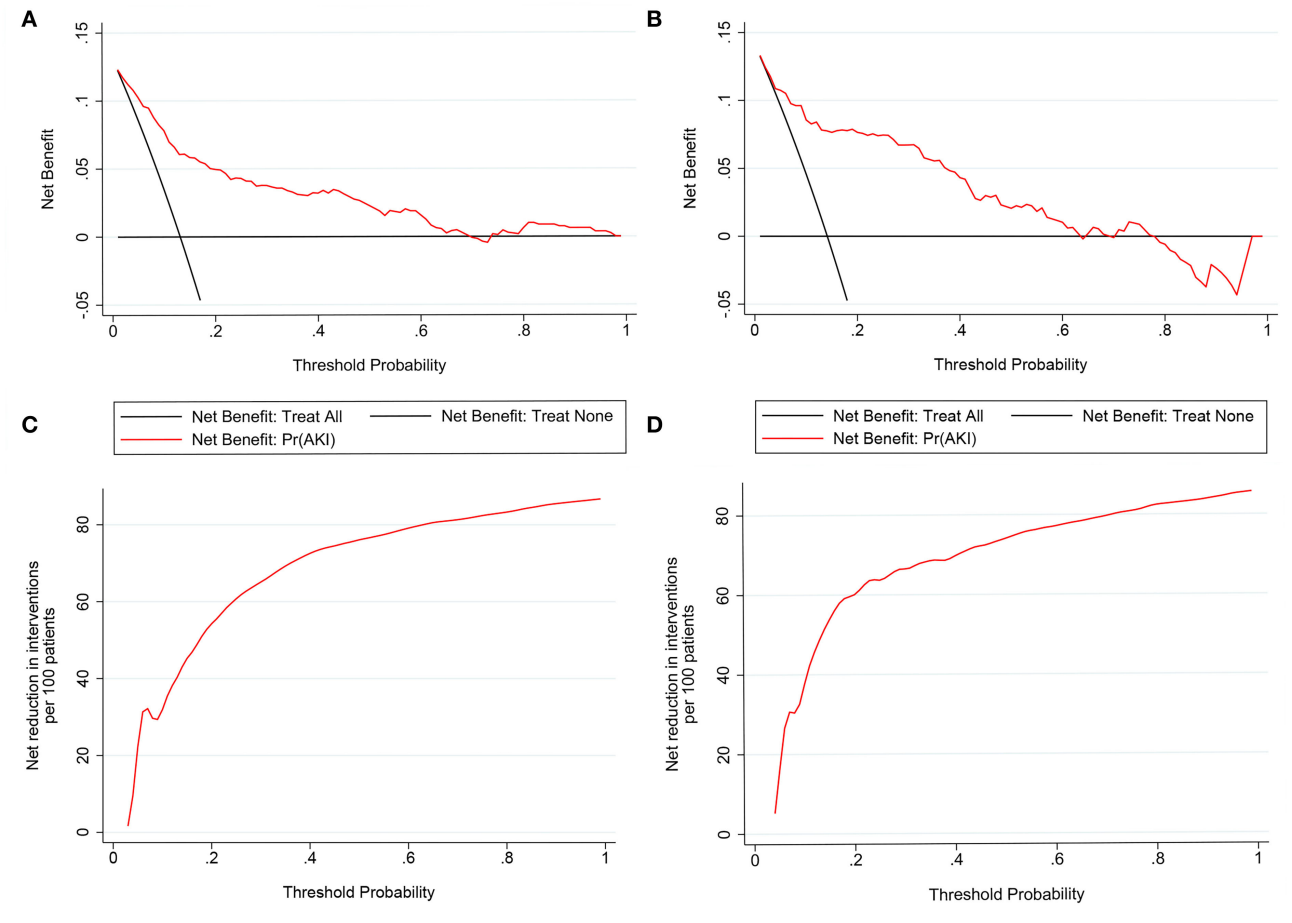
Decision curve analysis (DCA) of the nomogram in the training set **(A)** and the validation set **(C)**. The horizontal line indicates no patients develop acute kidney injury (AKI), and the gray oblique line indicates patients develop AKI. The blue solid line represents the AKI risk nomogram. In DCA, the nomogram shows a more net benefit than full or no treatment across a threshold probability range. Interventions avoided the curve of the nomogram in the training set **(B)** and the validation set **(D)**. The curve represents the net reduction in the number of interventions per 100 patients at a given threshold.

Nomograms showed good differentiating value for combined AKI in patients with AIS. The AUC values of AKI plots in the training and validation sets were 0.8529 (95% CI: 0.8036–0.8954) and 0.8598 (95% CI: 0.8017–0.8806), respectively (Figure 3). The calibration curves showed good calibration for both cohorts (Figure 4). In addition, the *p*-value of the Hosmer-Lemeshow goodness-of-fit test in the training set was 0.8814, and the corresponding value in the validation set was 0.4835. Both the calibration curve and the Hosmer-Lemeshow goodness-of-fit test indicated that the predicted results were highly consistent with the actual results. The DCA of the AKI nomogram was performed in both the training (Figure 5A) and validation sets (Figure 5B). The horizontal axis indicates that no one received the intervention, and the net gain was 0. The diagonal line indicates that everyone received the intervention. The net returns range from 0 to 12% and 0 to 13% in the training and validation sets when the prediction thresholds are in the range of 0–100% and 0–64%, respectively. The smaller the threshold, the greater the net gain. Interventions that avoided curves show a higher net reduction in interventions per 100 patients as the prediction threshold increases in the range of 0.01–1 in both the training (Figure 5C) and validation sets (Figure 5D). For example, in the training set, a prediction threshold of 0.24 reduces the intervention rate for patients by 60% without missing a diagnosis. The DCA and interventions that avoided curves visualize that the nomogram has significant predictive value.

## Discussion

This study predicts the short-term probability of AKI in patients with AIS by using routine information available in the ICU to construct a simple and easy-to-use nomogram. Patients with AKI receive antibiotic therapy and mechanical ventilation more frequently and have longer ICU stays, suggesting that patients with AKI typically suffer more adverse effects and financial burden. A previous study on patients with sepsis constructed a predictive model for AKI that included 5 indicators, such as baseline serum creatinine and glucose. The C index of the nomogram was 0.752, which had a good predictive performance. In addition, the prediction model was also developed to predict the risk of AKI in patients with diabetic ketoacidosis. The predictors used to construct the nomogram included diabetes, obesity, simplified acute physiology score II, bilirubin, creatinine, and BUN. The model has good discriminatory power, with area under the ROC curve values of 0.756 and 0.760 in the training and validation cohorts, respectively. Recently, Liu et al. constructed a glycemic-based predictive model for predicting the occurrence of AKI in stroke patients treated with endovascular treatment (EVT) to provide evidence for the glycemic management of patients (24). They screened stroke patients receiving EVT and included many glycemic-related factors, such as acute glycemia, hemoglobin bA1c (HbA1c), and average chronic glycemia, from the perspective of glycemic management. The study is more focused, especially on stroke patients with diabetes mellitus. However, it is important to construct a more generalizable predictive model for all patients with AIS. Therefore, our nomogram identifies variables that are easily accessible in the clinical setting. Moreover, the portability of the dynamic nomogram makes it possible for clinicians to predict the probability of AKI in patients with AIS at any time. The construction of the nomogram was performed well in terms of predictive performance, calibration, and clinical application, providing valuable information for selecting individualized treatment plans for different patients. More importantly, it bridges the gap between the high incidence of AKI after ischemic stroke and the absence of credible predictive modeling.

Our results showed that the probability of AKI in patients with AISS was 13.43%, with an incidence similar to that reported in previously published studies (25–27). Based on a meta-analysis in the United Kingdom, the combined incidence of AKI after ischemic stroke was 9.62% (28). Two pathways typically exist to influence the development of AKI after brain injury, such as neuroendocrine pathways, along with inflammatory and immune pathways. After a stroke, the hypothalamic-pituitary-adrenal axis activates, stimulating the adrenal glands to release glucocorticoids into the bloodstream (29). Prolonged high levels of hormonal state impair glomerular and tubular function. In addition, the sympathetic excitation that accompanies a stroke promotes the release of catecholamines from the adrenal glands, resulting in increased norepinephrine levels. Although the hyperadrenergic state facilitates the delivery of adequate oxygen to damaged tissues, and renal vasoconstriction, decreased renal perfusion, and reduced glomerular filtration rate appear to be unavoidable (30). Various studies *in vivo* and *in vitro* suggest that the renin-angiotensin-aldosterone system (RAAS) may play an important role in the development of kidney injury (31). Furthermore, the autoregulatory function of the kidney is impaired after stroke. Studies have shown a significant correlation between a decrease in estimated glomerular filtration rate (eGFR) and a decrease in autoregulation in the brain (32). Secondary alterations, such as hemorrhagic transformation and the presence of cerebral edema, may lead to worse stroke outcomes. Similarly, they have also been shown to be connected with reduced eGFR and poorer cerebral autoregulation (33). On the other hand, the compromised blood-brain barrier allows inflammatory cells that enter the brain to enter further into the systemic circulation, thereby causing secondary systemic inflammation (30). Infiltration of immune cells (M1-type macrophages) and activation of inflammatory factors (C-reactive protein, interleukin (IL)-6, IL-1β, reactive oxygen species [ROS], etc.) are important factors in the development of AKI (34).

Logistic regression analysis showed that BUN, creatinine, RDW, HR, the use of vancomycin, contrast agents, mannitol agent, the history of CHF, and OASIS were significantly associated with the occurrence of AKI in patients with AIS. AKI is characterized by a rapid decline in renal function or the development of significant renal failure accompanied by a significant increase in BUN and creatinine (35). Despite being susceptible to interference by age, sex, race, and weight, BUN and creatinine remain the most commonly used biomarkers of impaired kidney function. Studies have noted that mild increments in BUN and creatinine are associated with an increased incidence of AKI in patients in the ICU. In addition, patients with higher creatinine typically have longer hospital stays and higher mortality rates (36). In addition, it is inevitable that stroke patients need to undergo examinations with contrast agents, such as iodinated alcohol. Contrast-induced AKI is a very common cause of hospital-acquired AKI and usually occurs within 48–72 h after contrast injection (37). Although the mechanism is not fully understood, contrast agents eventually lead to epithelial and endothelial cell apoptosis, which in turn causes a decrease in glomerular filtration rate. Adequate blood volume expansion and the use of hypotonic contrast media can help to prevent AKI (38). CHF leads to complex interactions between the heart and the kidneys (39). Patients with a history of CHF are usually at increased risk of AKI due to inadequate renal reserve function and reduced renal perfusion (40). Moreover, our study shows that patients with AIS who develop AKI are usually associated with a higher HR. An excessively fast HR decreases cardiac output per beat, which decreases renal blood perfusion and fosters the development of AKI (41). Mannitol is an osmotic diuretic that is commonly used to relieve cerebral edema after cerebrovascular accidents. Despite conflicting data, mannitol is still commonly used in clinical practice to prevent AKI (42). Fisher et al. showed that patients' renal function is preserved when mannitol is administered in open-heart surgery, which may be associated with reduced cell swelling and deposition of cellular debris in the renal tubules (43). Further studies are needed in the future to determine the specific mechanisms by which mannitol is used to prevent AKI. OASIS reflects the severity of the patient's condition and may be an independent predictor of mortality 1 year after ICU admission. Similar to previous reports in the literature (44), our study showed that patients in the AKI group had higher OASIS scores than the patients with non-AKI and that higher OASIS scores were associated with a higher risk of developing AKI during ICU. RDW has value in predicting AKI in patients treated in a coronary care unit and in patients undergoing initial percutaneous coronary intervention (45, 46). The underlying mechanisms between RDW and AKI have not been completely elucidated, and the levels of systemic inflammation and oxidative stress in the kidney and systemic nutritional status reflected by RDW may be related to the development of AKI (47).

Here we give an example to illustrate how to use a nomogram. Suppose a patient with AIS, with a history of CHF, who was treated with vancomycin and mannitol during the treatment period, had an HR of 90 beats per min, an RDW of 18, a BUN of 20 mg/dl, a creatinine of 1 mg/dl, and an OASIS score of 30. According to Figure 2, the score corresponding to each individual parameter is obtained from the first row (the “Point” axis). Finally, the overall score was calculated as the sum of points for all parameters [30 (contrast agents) + 15 (CHF) + 0 (mannitol) + 21 (vancomycin) + 35 (HR) + 21 (RDW) + 27 (creatinine) + 23 (BUN) + 20 (OASIS) = 192]. This score corresponded to a risk of developing AKI at an ~22.3% level. More notably, the dynamic nomogram eliminates this step of the calculation. All that is required is to manually enter the patient's various laboratory parameters, medical history, and treatment received, depending on the actual situation. A dynamic nomogram will automatically output predicted values for the occurrence of AKI.

There are some limitations to this study. First, the patients included in the study were from only one medical center from 2001 to 2012, and the selection bias of the population and the continuous updating of treatment regimens may lead to a decrease in predictive performance, and multi-center data may be needed to validate the accuracy of the model in the future. Second, the data in this study were collected within 24 h of ICU admission, where there may be differences in laboratory variables between the emergency department and the general ward. Therefore, further studies are needed to confirm the validity of our findings. Finally, we constructed the nomogram. Since the nomogram was constructed based on 13 indicators, the sensitivity of the model performance may be reduced if data for one or two indicators of a patient are missing.

## Conclusion

Blood urea nitrogen, creatinine, red blood cell distribution width, heart rate, Oxford Acute Severity of Illness Score, the history of congestive heart failure, the use of vancomycin, contrast agent, and mannitol agents were identified as risk factors for AKI in patients with AIS. Furthermore, a dynamic nomogram was developed based on multivariate logistic regression of these factors. This is a simple scoring method to predict the risk of AKI in patients with AIS and helps to identify and intervene in high-risk patients earlier.

## Data availability statement

The data analyzed in this study was obtained from the Medical Information Mart for Intensive Care III (MIMIC-III) Clinical Database, the following licenses/restrictions apply: To access the data you must be a credentialed user, complete the required training (CITI Data or Specimens Only Research) and sign the data use agreement for the project. Requests to access these datasets should be directed to PhysioNet, https://physionet.org/, https://doi.org/10.13026/C2XW26.

## Ethics statement

Ethical review and approval was not required for the study on human participants in accordance with the local legislation and institutional requirements. Written informed consent for participation was not required for this study in accordance with the national legislation and the institutional requirements.

## Author contributions

GZ: conceptualization, data curation, and writing—original draft. ZF: investigation. TJ: methodology. XX and JW: software and supervision. LC: validation. WY: writing—review and editing. All authors contributed to the article and approved the submitted version.

## Conflict of interest

The authors declare that the research was conducted in the absence of any commercial or financial relationships that could be construed as a potential conflict of interest.

## Publisher's note

All claims expressed in this article are solely those of the authors and do not necessarily represent those of their affiliated organizations, or those of the publisher, the editors and the reviewers. Any product that may be evaluated in this article, or claim that may be made by its manufacturer, is not guaranteed or endorsed by the publisher.

## Supplementary material

Supplementary material S1 Baseline characteristics of the training and validation set populations.

Supplementary material S2 Results of multiple covariance tests.
